# Decoding Pain: Next‐Generation In Vitro Systems for Mechanistic Insights and Drug Discovery

**DOI:** 10.1096/fj.202501025RR

**Published:** 2025-08-15

**Authors:** Dara Khosrowshahi, Liesbet Lagae, Johanna Bolander

**Affiliations:** ^1^ Department of Physics and Astronomy KU Leuven Leuven Belgium; ^2^ IMEC Leuven Belgium; ^3^ Berlin Institute of Health Centre for Regenerative Therapies (BCRT), Berlin Institute of Health at Charité Universitätsmedizin Berlin Berlin Germany; ^4^ Julius Wolff Institute for Biomechanics and Musculoskeletal Regeneration, Berlin Institute of Health at Charité Universitätsmedizin Berlin Berlin Germany

**Keywords:** analgesics, chronic pain, co‐culture, induced pluripotent stem cells, in vitro pain models, nociceptors

## Abstract

Chronic pain affects approximately 20% of the population, significantly impacting daily life and increasing psychosocial burden for patients due to the limited effect of analgesics in providing reliable pain relief. This clinical impediment is largely due to a limited mechanistic understanding of human pain pathophysiology, caused by the limitation of models to study human pain mechanisms. Further, the lack of reliable models to study human pain‐associated mechanisms hinders the screening and evaluation of pain‐related drugs and therapies, leading to significant obstacles in the development of pain medications without inducing unwanted side effects. More complex and physiologically relevant in vitro models provide an opportunity to study human cells and tissues in a controlled environment while replicating key aspects of the native human environment. Further, these models are ethically advantageous by serving the 3R principle and enable the direct study of human cells and their physiological environments, facilitating the development of translational findings. In this review, we present the key molecular mechanisms of the pain sensory process, highlight the bidirectional crosstalk between nociceptors and non‐neuronal cells at the peripheral and central nervous system levels, discuss the current in vivo models and their drawbacks, and explore strategies for human‐relevant modeling by generating human nociceptors in vitro through various differentiation protocols of induced pluripotent stem cells (iPSCs). We also review the state‐of‐the‐art of in vitro pain model systems, including their electrophysiological characterization, compartmentalization strategies, and the use of agonist and antagonist assays targeting specific ion channels and receptors to validate these models. Additionally, we examine pain coculture model strategies that more closely replicate in vivo peripheral and central microenvironments. Finally, we discuss the current limitations and future perspectives of enhancing the physiological relevance and predictability of in vitro pain models for the development of novel analgesics and deepening mechanistic understanding.

## Graphical abstract

Developing an in vitro pain model that mimics essential features of the in vivo pain circuit requires careful consideration of multiple components and choices. These include selecting the cellular population for the model, choosing from the origin of nociceptors and other cells that are cultured, deciding between 3D or 2D dimensions, designing the compartments and choosing appropriate materials, ensuring electrophysiological readouts and validation of the model through agonist and antagonist assays.

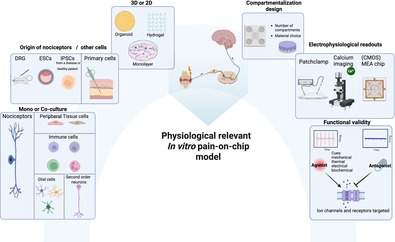

## 1 The Multi‐Faceted Nature of Pain

Pain constitutes an unpleasant sensory and emotional encounter linked to real or potential tissue harm, evolutionary developed to serve as a protective mechanism. Clearly, pain sensing allows an individual to retreat from hazardous situations upon sensing a harmful stimulus, but it becomes a significant burden during chronic conditions. The pain sensing mechanism is mediated by cellular signaling networks of the peripheral nervous system (PNS) which is processed by the central nervous system (CNS) [1]. Pain can be divided into two states: acute and chronic. Acute pain is a temporary sensation following tissue damage, irritation, or perturbation, such as after surgery or injury. In contrast, chronic pain is an enduring and dysfunctional state that heightens sensitivity to sensory stimuli, originating from irregular functioning of the nervous system, resulting in pain persisting well beyond the healing of the initial injury or pain inducing event, lasting for months or even years [2].

Chronic pain is a global phenomenon affecting over 1 in 5 people in the world [3]. Beyond the unpleasant sensation patients retain, the consequences of chronic pain are far‐reaching, including significant decrease in quality of life, work incapacity, loss of income, psychological disorders such as depression, an increased risk for suicide, anxiety, and substance addiction [4, 5]. Pain and its related disorders present high costs for global economies, with estimated direct and indirect healthcare costs of around $600 billion annually [6].

Chronic pain can be linked to different underlying pathologies and distinct clinical conditions like cancer, internal organ damage, neck/back/joint/muscle pain, and failed regeneration [5]. Further, chronic pain can be classified into two broad categories: nociceptive pain and neuropathic pain, both affected by the inflammatory status of the local and systemic environment [7]. While neuropathic pain refers to pain caused by lesions or diseases affecting the sensory nervous system [8], nociceptive pain represents the response to a noxious stimulus. The mechanisms between these two pains differ, and due to the prevalence of nociceptive pain and the potential of human in vitro models in this domain, it will be the focus of this review [9]. Specifically, nociceptive pain arises from defined tissue damage, irritation, or dysfunction, for example, skin burns, muscle strains, bone fractures, or joint trauma [8]. One of the common and significant causes of chronic nociceptive pain is osteoarthritis (OA) [10], a degenerative disease affecting over 590 million adults worldwide, characterized by joint inflammation, chronic pain, limited mobility, and decreased quality of life [11]. While healthy articular cartilage is avascular and aneural (characteristics that support frictionless and pain‐free joint movement), one of the hallmark features of OA pathology is the aberrant vascularization and innervation of the degenerating cartilage, contributing to nociceptive pain, and thus chronic pain in OA patients due to a lack of disease‐modifying therapeutics [12].

In chronic pain‐related diseases such as OA, nociceptive pain presents a multifaceted interplay of psychological and physiological elements, engaging a variety of cells and tissues. This complexity represents a major challenge for scientists and clinicians aiming to develop effective treatments for patients. Despite extensive research efforts, holistic mechanisms underlying pain remain elusive, resulting in persistent prevalence of chronic pain among patients due to poor treatment options.

## 2 Current Treatment Alternatives

To tackle chronic nociceptive pain, healthcare professionals aim to identify and treat the cause, which is often complex and therefore results in prescribed pain‐relieving treatments focused on managing symptoms rather than achieving a cure. Wearable medical devices as treatment are currently increasing in popularity, including transcutaneous electrical nerve stimulation to treat OA‐associated pain [13]. However, the use of wearables remains a niche, and the technology is still in development and hampered by side effects due to non‐targeted stimulation of nerve fibers [14]. That said, emerging afferent current stimulation techniques show promise by enabling more precise targeting of nerve pathways [15]. Nevertheless, the market for these devices remains small compared to the pain drug treatment sector, valued at approximately $7 billion versus $78 billion, respectively [16, 17].

Pharmacological approaches dominate the pain management landscape. Analgesics, that is, pain relief drugs, include both non‐opioid and opioid alternatives. Non‐opioids are generally over‐the‐counter drugs such as paracetamol (acetaminophen) or non‐steroidal anti‐inflammatory drugs (NSAIDs) like aspirin, targeting and inhibiting the cyclooxygenase (COX) enzymes involved in the synthesis of the inflammatory mediator prostaglandin during the inflammatory process of painful events [18]. The efficacy of non‐opioids has been proven for mild to moderate pain, but they do not provide sufficient relief for more intense pain and are associated with side effects such as gastrointestinal disorders like ulcers and, in case of overdose, acute liver failure [19].

Opioids are typically the next line of prescribed drugs to address severe pain and include substances ranging from legally prescribed pain relievers like oxycodone, codeine, and morphine to illicit drugs like heroin and fentanyl [20]. Opioids function by targeting specific opioid receptors on nociceptors, causing a disruption of the pain signals between the PNS and CNS [21]. This results in the patient experiencing a reduction in the sensation of pain. However, opioids not only affect nociceptive circuits but also other signaling circuits expressing opioid receptors, including the CNS and therefore causing a range of adverse effects like personality change, dysphoria, euphoria, and respiratory and cardiovascular disorders [22]. Further, clear evidence indicates that patients taking opioids long‐term are at an increased risk of opioid tolerance and dependence, with a significant risk of overdose. Despite these risks, opioids continue to be heavily lobbied and prescribed, contributing to an ongoing opioid addiction epidemic with millions of deaths associated [23, 24].

Consequently, the quest for effective pain medication alternatives without significant undesirable side effects persists. However, the current consensus related to mechanistic research and drug development states that the challenge lies not in the scarcity of pain drug candidates but in their complexity and reliability of testing. As a result, targeting specific peripheral nervous tissue could potentially achieve an elevated safety and efficacy profile compared to targeting the entire body [25]. To achieve this, innovative and sophisticated models to study and test the efficacy on peripheral neurons and their local environment are needed to develop new pain drugs to meet the clinical need.

## 3 The Role of the Nervous System in Pain Sensitization

The pathways involved in the nociceptive pain mechanism include local tissues and organs throughout the body where sensitization occurs, the PNS, which comprises neuronal cell bodies and nerves that extend from the spinal cord to innervate various parts of the body and transmit pain signals, and the CNS, consisting of the brain and spinal cord, which integrates these signals through neural circuits, modulates the perception of pain, and coordinates appropriate responses [26]. The nervous system's fundamental unit is the neuronal cell, firing electrical signals and transmitting them via synapses upon activation. Within the CNS, neurons form complex circuits and networks. Conversely, in the PNS, neurons are organized into ganglia containing cell bodies and nerve bundles, surrounded by connective tissue. Among PNS neurons are sensory neurons, specialized in sensing external stimuli such as heat, light, pressure, and pain. These sensory neurons constitute the afferent nerves of the PNS, and their sensitization allows them to transmit the message of external stimuli to the CNS for processing of the information [27].

### 3.1 Pain Sensing Nociceptors

Nociceptors are specialized sensory neurons responsible for transducing noxious stimuli into action potentials (AP) enabling pain sensitization, and two main classes of nociceptors exist: small diameter myelinated and unmyelinated sensory neurons. The myelinated are known as Aδ nociceptors and transmit fast and intense pain due to myelin (a lipid‐rich material) insulation that enhances conductivity. The small‐diameter unmyelinated sensory neurons are known as C nociceptors and encode slow aching pain; they represent the majority of the nociceptors in the PNS [28]. Nociceptors have a pseudounipolar structure, where their axons bifurcate into two branches: a peripheral and a central branch. The peripheral branch terminates in free nerve endings and densely innervates peripheral tissues such as the skin, the joints, the respiratory system, and the gastrointestinal tract [29]. Nociceptors can respond to mechanical, biochemical, and thermal noxious stimuli or any kind of stimuli on these tissues by converting them into electrical signals through a process called transduction [30]. The cell bodies of nociceptors are hosted in the dorsal root ganglion (DRG), while their central branches of the axon continue to carry the electrical signal and terminate in the dorsal horn (DH) of the spinal cord. Here, nociceptors form synapses with second‐order DH neurons to transmit pain signals within the CNS, leading to the perception of pain in the brain [30]. The current pharmacologic treatments can act at multiple points along the pain pathway, including targeting the transduction at the peripheral tissue level, the transmission in the spinal cord, or the perception step in the brain [31]. Specifically, one crucial aspect is to distinguish nociception, which denotes the electrical signal propagation and modulation in the nervous system, from pain, which represents the final perception of the unpleasant experience [32].

### 3.2 Peripheral Pain Molecular Mechanisms

The pain signaling stimulus causes an activation of the free endings of the nociceptors in the local pain environment, leading to an alteration of the membrane potential and subsequent AP generation. AP generation can occur through two primary interconnected mechanisms: (1) activation due to noxious stimuli via ion channels or (2) modulation via inflammatory mediators and receptors, both resulting in a flux of cations across the membrane causing the AP generation. Subsequently, if the membrane potential reaches a certain threshold, the initial signal is amplified and transmitted along the axon to the spinal cord, resulting in pain perception in the brain [33].

**FIGURE 1:**
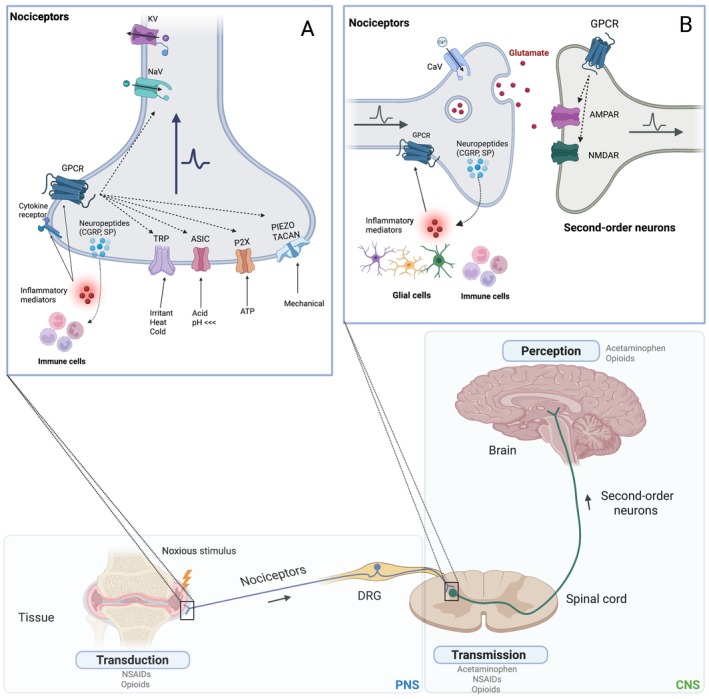
Nociceptive pathway and pharmaceutical interventions that modulate the signal at each stage, inspired by [31] (A) Representative ion channels and receptors responsible for transduction in nociceptors peripheral ending in tissues; (B) Representative ion channels and receptors responsible for transmission in spinal cord.

Nociceptors encode the initial pain stimuli into electrical signals through an array of ion channels at their endings in peripheral tissues, with each channel responding to different types of noxious stimuli (Figure 1A). The transient receptor potential (TRP) family includes TRPV1, an ion channel activated by noxious heat and capsaicin while TRPA1 and TRMP8 channels sense noxious cold and chemical compounds like menthol, allyl isothiocyanate or cinnamaldehyde [34].

Acid‐sensing ion channels (ASIC) represent another family of ion channels for nociception and are activated in low pH conditions (Figure 1A). Physiologically acidic conditions can occur due to low blood oxygen, fatiguing exercise, or inflammatory conditions [35]. Nociceptive pain can also be elicited by mechanical stimulation, causing deformation of the cellular membrane of the nociceptor, which activates a cellular response by regulating the flux of cation through mechanosensitive ion channels [36] (Figure 1A). Piezo1 and Piezo2 have recently been identified as specific mechanosensitive ion channels [37], where Piezo1 is more expressed in mechanically stimulated tissue cells (e.g., muscle [38], bone [39] or cardiac cells [40]), while Piezo2 is more prominent in DRG sensory neurons [37]. Further, recent discoveries highlight the essential role of TACAN ion channels in a certain subset of nociceptors, as TACAN channels appear to be more sensitive to high‐threshold painful mechanical stimuli, contrasting with Piezo ion channels, which have been identified for lower threshold stimuli [41]. It is also reported that nociceptors have adenosine triphosphate (ATP)‐responsive ion channels from the purinergic receptor (P2X) family, with the P2X3 subtype commonly found in sensory neurons. The P2X family of channels can be activated by ATP releases from the cytosol of damaged non‐neuronal cells due to stress, including mechanical deformation, during inflammatory states or injury environments (Figure 1A) [42].

Upon the transduction of a stimulus and a certain threshold of membrane potential has been reached, voltage‐gated ion channels become activated. These include voltage‐gated sodium (NaV) channels, with nine subtypes (NaV1.1‐NaV1.9) that depolarize the axonal membrane by allowing Na^+^ ions to flow into the nociceptors through the open channels (Figure 1A). Subtypes NaV1.7, NaV1.8, and NaV1.9 are predominantly expressed in nociceptors, while the others are expressed by the CNS (NaV1.1‐NaV1.3 and NaV1.6), skeletal muscle (NaV1.4) and in heart muscle cells (NaV1.5) [43]. After membrane depolarization, voltage‐gated potassium (KV) channels restabilize the membrane potential by allowing K^+^ ions to flow out of the nociceptors through the open channels (Figure 1A). NaV and KV channels are present throughout the axons of nociceptors and play a crucial role in amplifying the initial cation influx generated by the primary transducers upon stimuli, thereby propagating AP to the spinal cord [44, 45].

After transduction, the modulated nociceptive signal is relayed to the DH of the spinal cord, where it undergoes the transmission step to second‐order DH neurons. During the transmission step (Figure 1B), voltage‐gated calcium (CaV) ion channels located at the presynaptic terminals of nociceptors in the spinal cord regulate the AP‐mediated release of neurotransmitters into the synapse. The primary neurotransmitter of nociceptors is glutamate, causing an activation of glutamate receptors NMDA receptor (NMDAR) and AMPA receptor (AMPAR) on second‐order neurons, leading to an AP that relays the nociceptive signal to the CNS. Second‐order neurons are also indirectly activated by glutamate bonding to G‐protein‐coupled receptors (GPCRs) which subsequently activate NMDAR and AMPAR via intracellular pathways [46].

**FIGURE 2:**
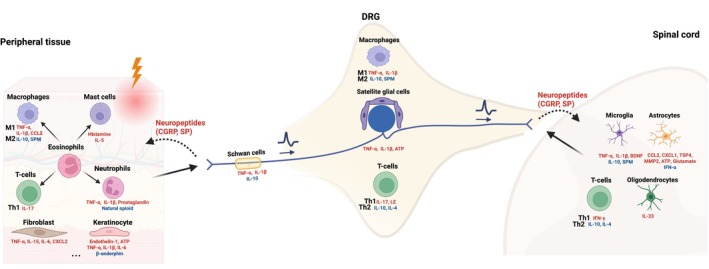
Pro‐inflammatory mediators (red) and anti‐inflammatory mediators (blue) produced by non‐neuronal cells in the nociceptive circuits, inspired from [30, 47]; tumor necrosis factor‐α (TNF‐α), interleukin (IL), interferon‐γ (IFN‐γ), leukocyte elastase (LE), specialized proresolving mediator (SPM), brain‐derived neurotrophic factor (BDNF), Thrombospondin‐4 (TSP4), Matrix Metallopeptidase (MMP2), c‐c motif chemokine ligand 2 (CCL2), c‐x‐c motif chemokine ligand 2 (CXCL2).

Inflammation plays a key role in the modulation of nociception, especially in chronic pain conditions [47]. This process involves various non‐neuronal cells including pro‐inflammatory immune cells, peripheral tissue cells, and glial cells, located throughout the nociceptive circuits including the peripheral tissue, DRG, and spinal cord levels. These non‐neuronal cells interact with nociceptors and contribute significantly to inflammatory responses (Figure 2). Specifically, the local immune cells at the peripheral tissues recognize and defend the body against harmful events such as injury. The peripheral tissue hosts resident or recruited immune cells at the injury site including macrophages/monocytes (M1 and M2), neutrophils (N1 and N2), mast cells, T‐cells (Th1, Th2, Tregs, cytotoxic T‐cells), all indirectly modulated by eosinophils, that release bursts of pro‐inflammatory mediators including growth factors, histamine, lipids (e.g., prostaglandin), cytokines (e.g., tumor necrosis factor‐α (TNF‐α), interleukin (IL)‐1β), and chemokines (e.g., c‐c motif chemokine ligand 2 (CCL2)) [47]. Additional cells present in the local peripheral tissues also release pro‐inflammatory mediators, such as damaged keratinocytes in the skin epidermis releasing Endothelin‐1, ATP, TNF‐α, IL‐1β, IL‐6 [48, 49] and synovial fibroblasts in the knee joint affected by OA have also been shown to sensitize nociceptors by an increased secretion of cytokines TNF‐α, IL‐15, IL‐6, and c‐x‐c motif chemokine ligand 2 (CXCL2) [50, 51]. Further, glial cells perform crucial functions to maintain the homeostasis of the nervous system, such as Schwann cells that provide support and insulation to neuron axons by producing myelin and also have a pro‐inflammatory effect via cytokine release including TNF‐α and IL‐1β.

Upon release of pro‐inflammatory mediators from non‐neuronal cells, they bind to their corresponding receptors on nociceptors such as the GPCR family or corresponding cytokines/chemokines receptors (Figure 2). This binding leads to activation of specific ion channels via intracellular pathways such as phosphorylation of ion channel proteins, lowering their activation threshold, and thus indirectly triggering a nociceptive signal, a process called peripheral sensitization (Figure 2) [30, 52, 53].

In chronic conditions, peripheral sensitization of nociceptors following tissue inflammation may induce activation of satellite glial cells and immune cell infiltration surrounding the DRG, which release pro‐inflammatory mediators such as TNF‐α, IL‐1β, ATP, and sensitize the cell body of nociceptors (Figure 2). Further along the nociceptive circuit in the CNS, the persistent peripheral inflammation induces immune infiltration of T‐cells and activation of glial cells in the spinal cord. These glial cells include microglia, acting as immune cells in the CNS; astrocytes, providing nutrients to nervous tissue and maintaining extracellular ion balance; and oligodendrocytes, supporting and insulating neuronal axons by producing myelin in the spinal cord, similar to the role of Schwann cells. During inflammatory conditions, glial cells are activated and release pro‐inflammatory mediators such as TNF‐α, IL‐1β, BDNF, leading to local inflammation and sustaining chronic pain by central sensitization (Figure 2) [30, 54].

Under homeostasis, non‐neuronal cells also release anti‐inflammatory mediators like the cytokines IL‐10, IL‐4, or molecules called specialized pro‐resolving mediators (SPM) along the nociceptive circuits to resolve inflammation. These mediators reduce nociceptive signals by targeting GPCR and cytokine receptors, causing both the action of the anti‐ and pro‐inflammatory mediators to modulate the nociceptive signal and the perceived pain (Figure 2). The dynamic inflammatory process is thus modular, and local non‐neuronal cells have divergent actions via mediator's release. However, in pathological conditions, a dysregulation of this dynamic pro‐ and anti‐inflammatory balance further drives chronic pain [55].

Upon nociceptor activation by noxious stimuli, they release neuropeptides such as calcitonin gene‐related peptide (CGRP) and substance P (Figure 2) [47]. These neuropeptides, in turn, influence immune cell activity by recruitment and triggering the release of pro‐inflammatory mediators from immune cells like monocytes and mast cells, thus sustaining inflammation through a positive feedback loop called neurogenic inflammation [47]. Interestingly, CGRP and substance P neuropeptides have also been identified to promote tissue repair mechanisms [56, 57, 58, 59]. Therefore, the effects vary depending on the context and arise from a complex interaction, likely influenced by the context of homeostasis and pathological state.

Stimuli via ion channels and modulation via inflammatory mediators and receptors are both essential for signaling danger to the body. However, sustained or intense exposure to noxious stimuli increases the reactivity of nociceptive nerves, leading to persistent peripheral and central sensitization, causing a trigger of signaling pathways that promote a long‐term shift in the activity of nociceptive circuits by higher expression of certain ion channels and receptors [60]. Indeed, transduction of noxious stimuli into AP is facilitated, and this may occur through either increased response to suprathreshold stimuli or reduction in the AP threshold. Clinically, this translates in pathophysiological conditions of respectively hyperalgesia, that is, exaggerated response to painful stimuli, and allodynia, that is, pain elicited by normally nonpainful stimuli [52]. Clinical observations have indicated that prolonged pain hypersensitivity at the peripheral tissue level induces structural changes of the corticolimbic brain region over time, contributing to the onset of chronic pain [61, 62].

## 4 In Vivo Models to Study Pain Pathophysiology

The lack of clinically relevant models to study human pain mechanisms is a major reason for the lack of effective pain therapies. This is strongly linked to a limited comprehensive understanding of pain mechanisms. Human pain can be studied in the clinical setting by limited means and presents numerous practical challenges. Further, it is inherently subjective and is constrained by ethical considerations [63]. Consequently, the majority of mechanistic research and drug screening involves the use of in vivo animal models, particularly rodents, to investigate pain [64]. Conducted experiments in terms of drug development typically include behavioral tests to assess an animal's ability to respond to a pain‐like stimulus, usually induced by heat, mechanical load, or electrical stimulus (Table 1). These tests can be used to measure the efficacy of new analgesics by evaluating whether pain sensitization is reduced upon drug administration. Apart from being time‐consuming and expensive, this poses a significant ethical issue as these experiments are carried out on unmedicated animals, causing severe suffering [65, 66]. Additionally, pain‐related research in animal models relies on indirect assessments of pain, such as pain‐like behavior, and primarily focuses on acute conditions, failing to adequately represent chronic human pain [67].

**TABLE 1:** Current standard behavioral tests for pain assessment in the clinical setting (adapted and expanded from [65, 66, 67]).

Test	Stimulus	Measurement
Tail‐flick	Heat	Apply thermal radiation to tail; measure tail movement
Hargreaves	Heat	Apply thermal radiation to paw; measure paw withdrawal
Hot plate	Heat	Place on hot plate; Measure paw reaction time
von frey	Mechanical	Apply filaments to inflamed area; Measure paw withdrawal force
Mechanical paw pressure (Randall‐Selitto)	Mechanical	Apply increasing force; measure pain behavior (withdrawal, struggling)
Facial Grimace Scale	Various	Elicit with acute pain stimulus; assess facial expression; Score based on orbital tightening, nose, ears, cheeks
Ultrasonic vocalization	Various	Elicit with acute pain stimulus; assess ultrasonic vocalizations
Nesting behavior	Various	Elicit with acute pain stimulus; assess nest building and integration time
Burrowing behavior	Various	Elicit with acute pain stimulus; assess burrowing behavior
Tail Clip (Haffner's)	Mechanical	Apply clip to tail; Measure tail movement reaction time
Electrical stimulus (flinch/jump)	Electrical	Apply electrical stimulus; measure flinch/jump response

Alternative models to study chronic pain in a more relevant physiological setting include creating an injury by experimental surgery or induction by chemical or biological agents to mimic certain disease states, as further outlined in Table 2. In these models, pain is measured by evaluating behavioral changes via pre‐defined tests (Table 1) and/or physiological changes in the animals pre‐ and post‐procedure, including for example, increased levels of pro‐inflammatory mediators in the affected tissue or histological alterations in local tissues [64, 69]. However, the pain and suffering in these animal models are even more sustained due to their chronic aspect.

**TABLE 2:** In vivo injury pain models in animal models (adapted and expanded from [63, 68]).

Model	Induction method
Joint injury model	Surgery (destabilization of the medial meniscus, anterior cruciate ligament transection, medial meniscal transection)
Incisional pain model (mimics postoperative pain)	Surgery, incision on skin or muscle tissue
Chronic pancreatitis (mimics pain associated with pancreatic disorders)	Surgery (pancreatic duct ligation) or injection (caerulein)
Low back pain model (degeneration intervertebral disc)	Surgery, puncture or injection (complete freund's adjuvant [CFA])
Inflammatory pain model	Injection of chemicals in paw or joint (carrageenin, dextran, CFA, formalin, zymosan, lipopolysaccharide) or irradiation (ultraviolet‐B)
OA pain model	Injection of chemicals into joint (monoiodoacetate, streptococcal cell wall induced arthritis, collagen‐induced arthritis)
Capsaicin‐induced pain model	Injection of capsaicin into paw
Acetic acid writhing test	Parenteral injection of acetic acid: measurements of retraction of abdomen and stretching of hind limbs.
Cancer pain model	Injection of cancer cells to induce tumor growth

Rodents such as rats and mice have traditionally been the primary animals used in pain research, mainly due to similarities in the neuroanatomy and physiology across mammalian species [70]. However, significant differences between rodents and human pain physiology are evident, particularly in transcriptome analyses. Specifically, the genes expressed in DRG where nociceptors are located do not overlap between rodents and humans, leading to both quantitative and qualitative differences in the expression of receptors and ion channels, such as variations in the composition, physiology, and functionality of NaV1.8 and NaV1.9 channels [71, 72, 73]. Consequently, rodent models have failed to function as pre‐clinical models for clinical translation in the development of new safe pain drugs. In fact, their use is highly associated with the high failure rates of pain drugs in the clinical phases (phase I to approval) estimated at 95% [74]. These failures can be attributed to both adverse side effects and a lack of efficacy in humans, despite the apparent safety and effectiveness observed in rodent models [64]. Such setbacks challenge the drug development market as the cost for clinical phase trials may reach 100 million USD [75]. More generally, the controlled environment often used in animal studies that typically rely on inbred animals diverges from the heterogeneous nature of humans and the individual impact on chronic pain, further impeding the successful translation of research findings to the human population [76].

While in vivo models may stay essential for some toxicity and safety studies, human in vitro models are undoubtedly required, not only in the field of pain research models but across various domains. The growing endorsement from regulatory and funding agencies to create diverse microphysiological systems (MPS) or organ‐on‐chip models reflects a commitment to reducing reliance on animal testing [77] and underscores a paradigm shift in research methodologies. This enables researchers to have the ability to directly study human cells and tissue of interest, marking a significant advancement in developing more relevant and translational findings.

## 5 In Vitro Models of Pain

Sensory neurons, including nociceptors, can be isolated from mouse or rat DRG and have been widely used as an in vitro tool for research around PNS and in vitro pain modeling. The isolation and culture processes have been well documented and reported [78]. Further, human‐related pain mechanisms can also be studied in vitro by utilizing human DRG (hDRG) neurons to avoid species differences and mitigate failures in translation, but access to viable hDRG neurons remains limited due to suitable donor availability [1], as postmortem collection is required. This leads to issues and major limitations for patient stratification and studies in particular patient groups. In the past years, the possibility to utilize human stem cells provides a major contribution to elevated model systems and technologies in the field and the potential to advance research [71].

Stem cells can be divided into embryonic and adult [79], where embryonic stem cells (hESCs) are derived from pre‐implantation embryos in early development, making them pluripotent and thus having the crucial ability to differentiate into almost any required cell type, including cells of the CNS and PNS such as nociceptors [80]. In contrast, adult stem cells are present in adult tissues; their primary role is to regenerate the tissue in which they reside. These cells are multipotent, limiting their ability to differentiate, and are also more rare and difficult to identify [79, 81]. Stem cells, especially hESCs, when expanded in vitro, offer several advantages over somatic cells, including high availability, unlimited self‐renewal capacity, and pluripotency [82]. This has led to a significant impact on research, as hESCs provided researchers with increased flexibility and versatility [83]. However, the use of human embryos raises ethical concerns and is largely rejected by the community [84]. To overcome this, scientists developed a protocol to reprogram adult human fibroblasts into induced pluripotent stem cells (iPSCs) based on the introduction of pluripotency genes known as the Yamanaka factors (Oct3/4, Sox2, Klf4 and c‐Myc [85]) which led to full reprogramming of the cell and restoration of pluripotency. Unlike hESCs, iPSCs are artificially generated stem cells originating from somatic cells, making them a more ethically viable source, but they still allow for the study of patient‐specific characteristics as they maintain their plasticity during reprogramming. This also means that iPSCs can be personalized for each patient by collecting their own somatic cells, paving the way for more tailored treatments [86].

**FIGURE 3:**
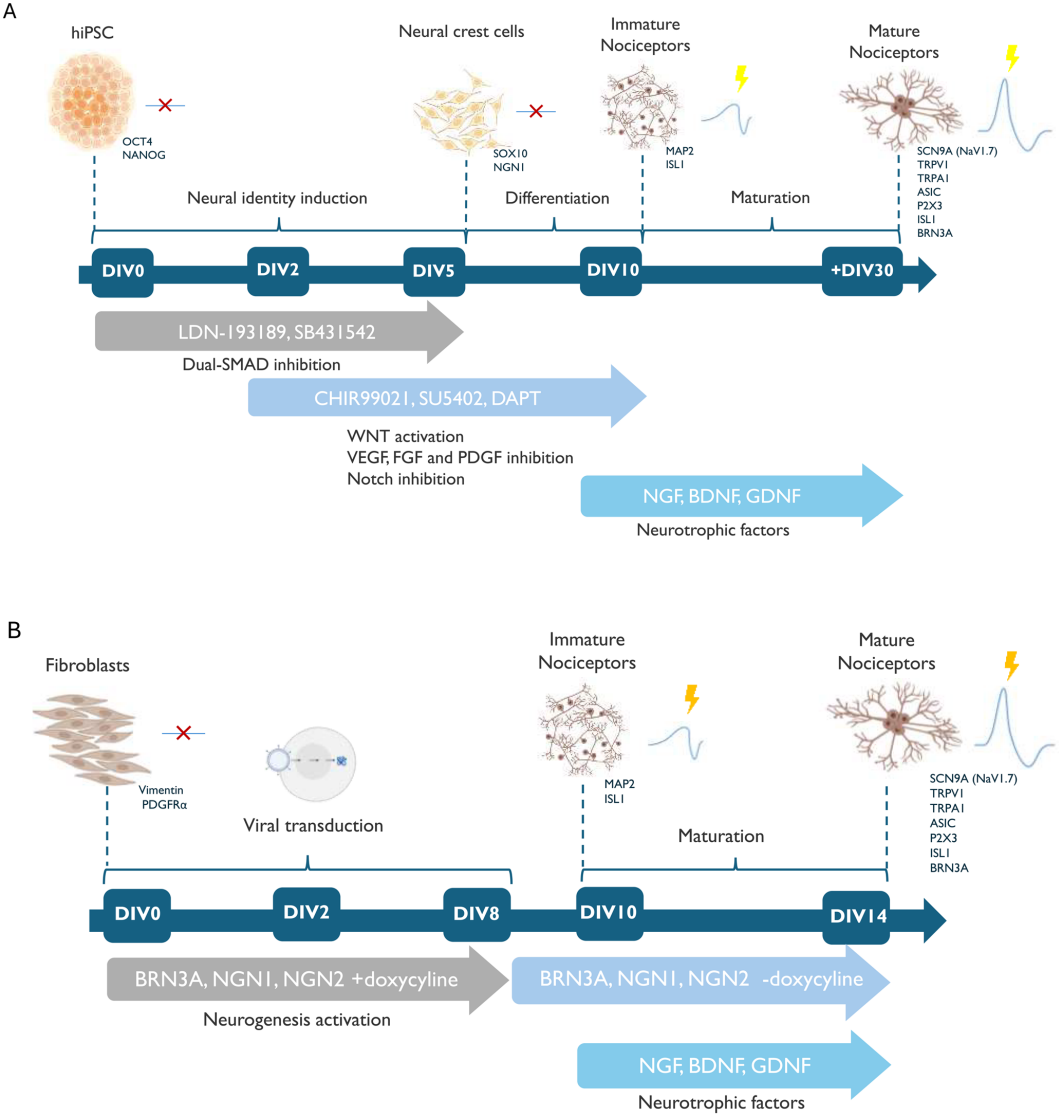
(A) The Chamber protocol of iPSCs differentiation into nociceptors involves several steps from neural identity induction to differentiation and maturation, with each step expressing specific biomarkers, inspired from [88]. First, neural identity is induced over 6 days using small molecule pathway inhibitors (SB431542, LDN‐193189). After 3 days, additional small molecules (CHIR99021, SU5402, and DAPT) are introduced to promote specific nociceptor differentiation. Following 11 days, neurotrophic factors are required for maturation, which can extend for an additional 20 days. (B) Direct reprogramming of fibroblast into nociceptors by Blanchard et al. [89]. Fibroblasts are first infected with lentivirus with the transcription factors BRN3A, NGN1, NGN2, transcription factors are induced by doxycycline. 7 days post induction, doxycycline was withdrawn. 10 days post‐induction, media was replaced with neural maintenance media, with the neurotrophic factors, DIV = day in vitro.

In pioneering work by Chambers et al. [87], a protocol for differentiating iPSCs into nociceptors was demonstrated for the first time by introducing five small molecules pathway inhibitors (SB431542, LDN‐193189, CHIR99021, SU5402, and DAPT) to induce neural identity and differentiation (Figure 3A) [87]. The first two small molecules, SB431542 and LDN‐193189, function as dual‐SMAD inhibitors to efficiently convert iPSCs into neural crest cells, inhibiting the bone morphogenetic protein (BMP) and transforming growth factor‐β (TGF‐β) signaling pathways, both of which utilize SMAD proteins for signal transduction (Figure 3A). The following molecules, CHIR99021, SU5402, and DAPT, accelerate neural crest specification and peripheral neuron formation from the neural crest cells by Wnt pathway activation and inhibition of Notch, vascular endothelial growth factor (VEGF), fibroblast growth factor (FGF) and platelet‐derived growth factor (PDGF) signaling pathways (Figure 3A). Upon differentiation, the cells display immature neuronal morphology with several neurites forming an arborised monolayer. Over 20 days, neurotrophic factors (brain‐derived neurotrophic factor (BDNF), glial cell line‐derived neurotrophic factor (GDNF), nerve growth factor (NGF), and ascorbic acid) helped neurons mature into ganglia‐like structures (Figure 3A) [90].

The differentiation protocol has since the first publication been followed by modified versions where the timing and nature of the small inhibitors vary depending on the specific experiment (Table 3). With these adaptations, different sensory neuron populations have been obtained, such as pruriceptors, which are sensory neurons for itching; proprioceptors that are the sensory neurons specialized in detecting the position and movement of our body parts; or mechanoceptors representing sensory neurons for the sense of touch and non‐noxious mechanical pressure [1]. Importantly, differences in the efficacy and reproducibility of each of the specific iPSCs differentiation protocols depend on their initial cell density and surface coatings as well as the type and genetic variations of the initial somatic cells [111], highlighting that the protocol needs to be selected between published studies, and thorough characterization is crucial.

**TABLE 3:** Differentiation protocols of iPSC‐derived sensory neurons (adapted and expanded from [72, 91, 92]) Note: ESCs protocols are not mentioned here.

Origin of sensory neuron	Neural identity induction	Sensory neuron differentiation	Maturation	Prevalent sensory neuron subtype	Characterization	References
*IPSCs differentiation*						
iPSC	SB431542 (D0‐D5) LDN192189 (D0‐D5)	CHIR990021 (D3‐D11) SU5402 (D3‐D11) DAPT (D3‐D11)	BDNF GDNF NGF (NT‐3) (cAMP, ascorbic acid) NGF	Nociceptors	Patch‐clamp Calcium imaging RNA‐seq Immunostaining Flow cytometry	Chambers et al. [87] Similar protocols with small changes: [93, 94, 95, 96, 97, 98, 99]
iPSC	SB431542 (D0‐D5) LDN192189 (D0‐D5)	CHIR990021 (D2D7) SU5402 (D2‐D8) DAPT (D2‐D8)	NGF GDNF BDNF NT‐3	Proprioceptors	Patch‐clamp qPCR Immunofluorescence Flow cytometry	Dionisi et al. [100]
iPSC	Noggin (D0‐D10) SB431542 (D0‐D10)	SB431542 (D11‐D18) EGF (D11‐D18) FGF(D11‐D18)	BDNF GDNF NGF NT‐3 ascorbic acid cAMP	Pruriceptors	Calcium imaging RT‐qPCR Immunocytochemistry Flow cytometry	Umehara et al. [101]
iPSC	Sphere Medium FGF EGF	Retinoic acid NGF BDNF GDNF NT‐3	n.a.	Mechanoceptors	Patch‐clamp Calcium imaging RNA‐seq in situ hybridization Immunocytochemistry	Schrenk‐Siemens et al. [102]
iPSC	SB431542 bFGF/EGF	BRN3A NEUROG2	BDNF GDNF NT‐3 NGF	Mechanoceptors	Patch‐clamp Calcium imaging RT‐qPCR Bulk and single‐nuclei RNA‐seq RNA in situ hybridization Immunocytochemistry	Nickolls et al. [103]
iPSC	SB431542 (D0‐D10) LDN192189 (D0‐D10)	CHIR990021 SU5402 DAPT	BDNF GDNF NGF NT3 ascorbic acid	Nociceptors Proprioceptors Mechanoceptors	Patch‐clamp combined with single‐cell‐RT‐PCR qPCR Bulk and single‐cell RNA‐seq Western blotting Immunocytochemistry Flow cytometry	Mazzare et al. [104]
iPSC	CHIR98014 (D0‐D3) A83 01 (D0‐D3)	CHIR98014 (D3‐D14) A83 01 CEPT DBZ PD173074	PD0332991 BDNF GDNF NGF NT‐3	Nociceptors	MEA Patch‐clamp Calcium imaging RNA‐seq in situ hybridization ELISA Immunocytochemistry	Deng et al. [105]
iPSC	CHIR99021(D0‐2) BMP (D0‐D2) Y‐27632 (D0‐2)	SB431542 (D2‐12) CHIR99021 (D2‐D12)	NGF GDNF BDNF NT‐3 DAPT retinoic acid	Nociceptors Proprioceptors Mechanoceptors	MEA RT‐qPCR Immunocytochemistry Flow cytometry Immunopanning	Saito‐Diaz et al. [106]
*Direct reprogramming*						
Fibroblast	n.a.	BRN3A NGN1 NGN2	NGF BDNF GDNF	Nociceptors Proprioceptors Mechanoceptors	Patch‐clamp Calcium imaging RT‐qPCR Immunocytochemistry	Blanchard et al. [89]
Fibroblast	n.a.	ASCL1 MYT1L NGN1 ISL2 KLF7	FGF BDNF CNTF GDNF NGF	Nociceptors	MEA Patch‐clamp Calcium imaging qPCR Single cell RT‐PCR ELISA Immunocytochemistry	Wainger et al. [107]
Multipotent somatic stem cells in hair follicles	SHH CHIR99021	LDN193189 DAPT NT‐3 HGF	n.a.	Nociceptors	Calcium imaging qPCR Immunocytochemistry Immunohistochemistry	Wilson et al. [108]
CD34^+^ hematopoietic cells (neural precursor cells (NPC) as intermediates)	SCF Flt‐3 L IL3 TPO cytokines SB431542 LDN193189 Noggin CHIR99021 bFGF EGF	DAPT SU5402 CHIR99021	BDNF GDNF NGF NT‐3	Nociceptors	Calcium imaging RT‐PCR Immunocytochemistry Flow cytometry Neurite length analysis HPLC	Lee et al. [109]
CD34^+^ hematopoietic cells (neural precursor cells (NPC) as intermediates)	OCT‐4 SB431542 LDN193189 CHIR99021 bFGF EGF	DAPT SU5402 CHIR99021	BDNF GDNF NGF NT‐3 Ascorbic acid Forskolin	Nociceptors	Calcium imaging Immunocytochemistry Flow cytometry Neurite length analysis	Vojnits et al. [110]

Differentiation of iPSCs into nociceptor typically requires beyond 20 days to gain their morphological and electrophysiological characteristics [87]. At the end of this maturation period, gene expression comparison between primary human (h)DRG sensory neurons and iPSC‐derived sensory neurons identified an 84% overlap of hDRG ion channel genes in iPSC‐sensory neurons [93]. Yet, the maturity level of iPSC‐derived sensory neurons remains lower than that of primary hDRG sensory neurons due to their smaller size and reduced electro‐responsiveness, resulting in an incomplete replication of the in vivo situation [72].

Following the revolution of iPSC generation, direct reprogramming of somatic cells using transcription factors into various differentiated cells, including sensory neurons, emerged as a more rapid method to generate and investigate sensory neurons (Table 3). In 2015, direct neuronal differentiation of human fibroblasts into nociceptors was generated. This was achieved by Wainger et al. employing five key transcription factors: ASCL1, MYT1L, NGN1, ISL2, and KLF7, while Blanchard et al. achieved their results with three: BRN3A, NGN1, and NGN2 (Figure 3B) [89, 107]. These protocols enabled the generation of sensory neurons in fewer steps, offering a faster differentiation strategy. However, a significant issue is the low conversion rates of somatic cells into nociceptors due to a high differentiation variability, preventing their scalability and widespread adoption compared to the initial Chamber protocol. In the same idea, Wilson et al. [108] succeeded in directly differentiating multipotent somatic stem cells that reside in the bulge of hair follicles into nociceptors via a small molecules strategy, thereby avoiding genetic changes via transcription factors. However, here too, high differentiation variability remains, indicating the continuing need for an improved process [108]. Further, direct reprogramming has been established using different somatic cells such as blood cells and incorporating an additional differentiation step involving neural precursor cells (NPC) as intermediates (Table 3) [109, 110], allowing for better differentiation control and reduced variability, thereby increasing the efficiency of nociceptor output while maintaining a more rapid process.

Clearly, the establishment of innovative differentiation and reprogramming methods paves the way for greater availability and access to nociceptors and the ability to study human pain sensing mechanisms with patient‐targeted pain in focus. Yet, further efforts are needed to close the gene expression gap between primary human nociceptors and the generated ones.

In addition to the generation of human nociceptors, iPSCs differentiation protocols for various other cell types involved in the nociceptive circuit have been developed, including for both immune cells: macrophages [112], neutrophils [113], mast cells [114], T‐cells [115], and peripheral/central glial cells: microglia [116], astrocytes [117], oligodendrocytes [118], Schwann cells [119]. Additionally, protocols for second‐order DH neurons of the spinal cord have been established [120]. These experimental protocols enable the development of in vitro human models with enhanced physiological relevance and improved biomimetic cultures. Ultimately, they facilitate more advanced studies of nociceptors cultured with supporting cells known to play crucial roles in human physiological pain sensitization at both peripheral and central levels.

### 5.1 Electrophysiological Characterization

The functional characterization of nociceptors and physiological readouts is essential for the assay outcome. Nociceptors are excitable cells; consequently, they transition from a resting state to an excited state upon activation, and this transition enables the measurement of an extracellular potential via electrodes [121]. Further, this translates into spike signals that serve as signatures for neuronal AP. Tools for electrophysiological characterization of nociceptors are thus crucial in developing in vitro pain models to understand mechanisms and monitor the effects of induced stimuli or analgesics.

In vitro, whole‐cell patch‐clamp methods efficiently measure neuronal extracellular electrical activity in various modes, including current clamp, voltage clamp, dynamic clamp, and can be manual or automatic [122]. However, these measurements are time‐consuming, preventing the scalability of sensing sites, and the invasive nature disrupts the intracellular milieu and therefore omits the possibility for complementary readouts [123]. Calcium imaging represents an alternative with fluorescent markers used to record intracellular calcium flux of multiple cells simultaneously, indirectly linked to electrical activity, but is still limited in throughput, temporal resolution, and potential functional interference by the used dye [124].

**FIGURE 4:**
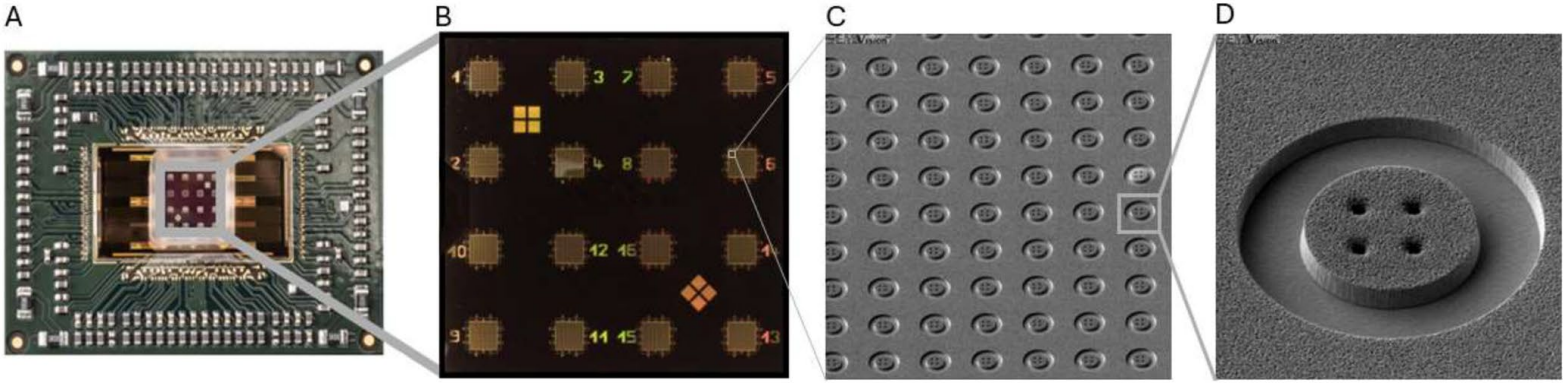
CMOS MEA chip architecture for extracellular recording (A) features integrated circuitry with 16 384 TiN microelectrodes organized into 16 clusters (B). Each cluster contains a 32 × 32 array of microelectrodes (C), which are further organized into pixels of 4 microelectrodes each of a size 8 μm with a 15 μm pitch (D). The subcellular size of the electrodes enables single‐cell resolution and the detection of electrical activity in individual nociceptor axons. This data can then be analyzed at the network level in terms of firing rate or visualized as spikes in a raster plot. Adapted from [126, 127].

An alternative technology on the rise is microelectrode arrays (MEA), offering a non‐invasive and label‐free method for measuring in vitro extracellular electrical activity. This technology enables rapid and dynamic electrophysiological characterization of the entire nociceptor population cultured on the chip, which can be pre‐coated with adhesion molecules to improve culture conditions. Depending on the design, MEAs can offer high throughput, high resolution, and limited operator expertise [125]. To achieve this, materials and systems integration is critical, and complementary metal‐oxide semiconductor (CMOS) technology in MEA chips has been shown to enable the integration of circuitry with above standard density while allowing for smaller electrodes with maintained signal‐to‐noise ratio, which is particularly relevant when culturing IPSC‐derived nociceptors to better detect their electrical firing. It has been demonstrated that these CMOS MEA chips can incorporate thousands of electrodes, reaching up to 16 384 microelectrodes (Figure 4) [128] and 26 400 microelectrodes [129, 130] compared to hundreds or below in classical MEA chips [131]. The increased electrode density and the subcellular sizes of the electrodes on these platforms enable experiments at high spatial resolution, enabling single‐cell resolution and the detection of electrical activity in individual nociceptor axons (Figure 4) [129]. Additionally, a higher sampling rate (i.e., the number of electrophysiology data points recorded per second) enables sub‐millisecond temporal resolution essential for monitoring neuronal AP [126, 128].

Furthermore, the development of CMOS MEA technology has allowed the integration of additional modalities on the same chip, such as impedance measurement offering new insight on cell attachment and mobility [128], and stimulation units that allow to simultaneously stimulate under applied voltage specific nociceptor axons and record their electrical activity [128, 131]. The performance of this technology positions it as a promising platform for evaluating nociceptor functionality, with high potential to unravel the complexities of nociceptive pain.

To further improve the replication of human biology, 3D MEA technology is being developed enabling it to interface with neurons in a 3D environment using a mesh structure [132, 133] or helical structure [134]. In comparison to 2D, these chips better mimic the in vivo environment by allowing neuritic outgrowth in space and facilitating the study of more physiological organoid models. Although this strategy enhances spatial resolution, broader adoption of this technology requires improved computational tools and a higher number of electrodes to accurately detect electrical activity changes in the tissue's third dimension [132].

### 5.2 Compartmentalization

A crucial aspect of co‐culture studies is the compartmentalization of various cell populations within the system using biocompatible materials, allowing cells to be cultured in a spatial arrangement similar to that of the in vivo circuit. Microchannel designs within these materials enable the sprouting of nociceptor axons into adjacent compartments, establishing a diffusion barrier but enabling communication with other cells. Meanwhile, the cell body of nociceptors is isolated from the axons to replicate their in vivo location in the DRG [135]. Moreover, the optimization of microfluidics enables the use of specific culture media for each cell population, providing improved control of their culture environment. The most popular material for this purpose is the elastomer polydimethylsiloxane (PDMS). The fabrication involves using soft lithography to create polymer molds with the desired design, followed by pouring and curing PDMS. It has been widely adopted due to rapid prototyping, low cost, optical transparency, biocompatibility for long‐term culture, and gas/O_2_ permeability [136, 137]. However, in addition to its limited scalability for high‐throughput manufacturing, a significant drawback is that small molecules like drugs and media supplements can permeate into the material, decreasing the intended concentration. This can compromise experimental reproducibility and potentially result in misrepresentation of drug candidate toxicity and efficacy. Promising alternative materials have been investigated, including thermoplastic polymers like cyclic olefin copolymer (COC) or poly(methyl methacrylate) (PMMA) that have shown improved ability to prevent small molecule absorption [138]. Advanced in vitro models often incorporate sensors and different substrates, requiring surface treatment (such as plasma, temperature and solvent treatment) to ensure seamless bonding [139, 140]. For example, bonding materials like silicon for MEA chips can be challenging due to incomplete bonding and delamination, leading to leakage. Other materials, like off‐stoichiometry thiol‐ene polymer, commercially called OSTEMER, allow for direct bonding without treatment with sealing to any substrate due to the presence of an excess of thiol or allyl groups on the surface [141]. These critical points clearly point out that currently used materials represent advantages and disadvantages, requiring careful consideration based on experimental needs.

### 5.3 State of the Art

By combining improved differentiation protocols for nociceptor generation with different electrophysiological techniques and materials for compartmentalization design, researchers now have powerful tools to develop physiologically relevant human in vitro pain models. A vast majority of studies and patents on different in vitro pain models can be found in the literature, including respectively monocultures of nociceptors (Table 4) as well as co‐cultures with other cell types (Table 5). Further, different assays used in these studies to test the electrophysiology of the nociceptors in the models exist and need to be selected carefully based on the aim. The utilization of human in vitro models allows for detailed perturbations including pain stimulus induction (agonists) and analgesic administration (antagonists) that target different ion channels and receptors involved in the transduction and transmission steps (Figure 1). This approach allows for tailored monitoring of compound efficacy on activating the AP of nociceptors or suppressing their firing, thereby validating and refining the model.

**TABLE 4:** Classification and results of studies on human in vitro pain models.

Monoculture model	Origin of nociceptors	Electrophysiology characterization	Pain stimulus/agonists		Analgesics/antagonists		Results and comments	References
3D organoid	hESC	MEA chip	*Targeting ion channels:* AITC (TRPA1) Capsaicin Velvet ant venom Heat		*Targeting ion channels:* ProtoxinII (NaV1.7)	*Targeting receptors:* Bicuculline (GABA receptor) MK‐801 (NMDA receptor) tetrahydrocannabinol (THC)	Increased neuronal firing to capsaicin, AITC, velvet ant venom Decreased neuronal firing with non‐opioid drugs ProTx‐II and THC	[142]
2D	IPSCs	Patch‐clamp and MEA chip	*Targeting ion channels:* Capsaicin ATP (P2X)		*Targeting ion channels:* Lidocaine	*Targeting receptors:* DAMGO (opioids receptor)	Increased neuronal firing with capsaicin and ATP measured by patch‐clamp Increased neuronal firing with ATP measured by MEA chips Neuronal firing silenced with the lidocaine and the opioid DAMGO measured by MEA chips	[98]
2D	IPSCs	MEA chip	*Targeting ion channels:* Capsaicin AITC + side effect of oxaliplatin (anticancer drug) Menthol (TRPM8) Heat		*Targeting ion channels:* AMG9810 (TRPV1) A967079 (TRPA1) AMTB hydrochloride (TRPM8)	n.a.	Increased neuronal firing with capsaicin, AITC, menthol and as the temperature rose from 37°C to 46°C Increased neuronal firing with the response to AITC increased in a concentration‐dependent manner with oxaliplatin, reproducing the heightened cold sensitivity in humans	[143]
2D	IPSCs	MEA chip	*Targeting ion channels:* Capsaicin Menthol Heat	*Targeting receptors:* Bradykinin Histamine (histamine GPCR) Chloroquine (GPCR)	*Targeting ion channels:* AMG9810 (TRPV1) ProTx‐II (NaV1.7)	*Targeting receptors:* PEAQX (NMDA receptor) NBQX (AMPA receptor) Pyrilamine (histamine GPCR)	Capsaicin, menthol, bradykinin, and heat (≥ 43°C) increased firing of nociceptor No response to capsaicin was observed after administration of AMG9810 Onset of bradykinin neural activity was longer than capsaicin and menthol PEAQX and NBQX reduced firing but also the number of bursts Pyrilamine reduced the neuronal excitability of histamine Chloroquine increased the firing ProTx‐II reduced the spontaneous firing	[144]
2D	Mice DRG and human OA patient synovial fluid	Calcium imaging and patch‐clamp	*Targeting ion channels:* Capsaicin Menthol KCl (CaV)		*Targeting ion channels:* Nifedipine (CaV) Tetrodotoxin (Nav) Ruthenium red (TRP) Amiloride (ASIC) APETx2 (ASIC3)	*Targeting receptors:* YM‐245890 (GPCR)	Administration of synovial fluid from human OA patient increased firing of mouse knee sensory neurons to capsaicin and menthol Synovial fluid from human OA reduced when administered with nifedipine and tetrodotoxin	[145]
3D spheroid	IPSCs	CMOS MEA chip	*Targeting ion channels:* Capsaicin Voltage stimulation		n.a.		Clustering of the nociceptor's population based on axonal electrical activity response to voltage stimulation	[129]
3D organoid	IPSCs	Calcium imaging	*Targeting ion channels:* Capsaicin		n.a.		Neurospheres with axonal bundles, unidirectionally elongated into microchannels during 62 days Capsaicin increased the firing in the neurosphere aswell as in the axon bundle	[146]
2D	hDRG obtained postmortem from organ donors both with no pain and pain history	Patch‐clamp	n.a.		n.a.		hDRG neurons were classified into three types: repetitive spikers, single spikers and burst‐firing neurons Repetitive‐spiking neurons expressed a higher expression of TRPA1 than in single‐spiking neurons Clusters based on electrophysiological data were linked to transcriptionally defined subpopulations of hDRG neurons Repetitive‐spiking neurons from pain donors showed hyperexcitability compared to those from no pain donors hDRG neurons from pain donors showed higher expression of Nav1.7 and NaV1.8 contributing to higher hyperexcitability	[147]
2D	Inherited erythromelalgia patient IPSCs‐derived	Dynamic patch‐clamp	n.a.		n.a.		Sensory neurons derived from two patients with inherited erythromelalgia, both carrying the same Nav1.7 mutation but exhibiting different pain levels (mild vs. severe), revealed that neurons from the patient with severe pain were more excitable Whole‐exome sequencing of the pain‐resilient patient uncovered a missense variant in the Kv7.3 channel (KCNQ3), suggesting this variant may play a role in pain resilience	[148]
2D	Inherited erythromelalgia patient IPSCs‐derived	Dynamic patch‐clamp	n.a.		*Targeting ion channels:* PF‐05089771 (NaV1.7)		Dynamic clamp can produce hyperexcitability in sensory neurons from inherited erythromelalgia patients with two different NaV channel mutations (S241T and I848T) They show that blockade of approximately 50% of NaV1.7 currents can reverse neuronal hyperexcitability to baseline levels	[149]
2D	hDRG	Patch‐clamp	n.a.		n.a.		Missense mutations in the Nav1.9 channel have been identified in individuals with painful peripheral neuropathy. These mutations depolarize the resting membrane potential of DRG neurons, enhance spontaneous activity, and increase evoked firing, contributing to heightened neuronal excitability	[150]
2D	Inherited erythromelalgia patient IPSCs‐derived	Patch‐clamp and MEA chip	Skin temperature (33°C), core body temperature (37°C), and non‐noxious warmth (40°C).		n.a.		Whole exome sequencing reveals that KV7.2 channels mutation (T730A) causes a gain of function of inherited erythromelalgia, and that this variant hyperpolarizes resting membrane potential and reduces excitability of iPSC‐SNs	[151]

**TABLE 5:** Classification and results of studies on coculture in vitro pain models.

Co‐culture model	Origin of nociceptor and cells	Electrophysiology characterization	Pain stimulus/Agonist		Analgesics/Antagonist		Results and comments	References
3D compartmentalized innervated skin (keratonicytes and fibroblasts)	hIPSCs nociceptors and human skin	n.a.	*Targeting ion channels*: Capsaicin		n.a.		Without skin construct, capsaicin administration caused more retraction of nociceptor neurites	[152]
3D compartmentalized innervated epidermal (keratonicytes)	Rat embryo DRG and human skin	Calcium imaging	*Targeting ion channels*: Administration in epidermal compartment: Capsaicin 4αPDD (TRPV4)		n.a.		Keratinocytes increase the firing of DRG neurons and induce a higher expression of TRPV1	[153] Similar studies [154] patent, [155] patent, [156, 157, 158, 159, 160, 161]
2D innervated vasculature direct cell–cell contact and compartmentalized	Mice DRG and mice endothelial cells	Calcium imaging	*Targeting ion channels*: Capsaicin low pH 5.4 (ASIC)		n.a.		Endothelial cells enhance sensitivity of nociceptors to stimuli	[162]
2D co‐culture with fibroblast‐like synoviocyte	Mice DRG and mice fibroblast‐like synoviocytes	Calcium imaging and patch‐clamp	*Targeting ion channels*: Capsaicin Menthol Cinnamaldehyde (TRPA1)		n.a.		Fibroblast‐like synoviocytes activated by TNF‐α enhance sensitivity of nociceptor to stimuli Enhanced TRPV1 function but downregulation of TRPA1 and TRPM8	[50]
3D compartmentalized innervated cartilage constructs	Mice DRG and human monocytes and chondrocytes	n.a.	*Targeting receptors*: Secretome of proinflammatory macrophages producing proinflammatory cytokines (IL‐6, TNF‐α, IL‐1β)		n.a.		Enhanced axonal growth in nociceptors with pro‐inflammatory macrophage secretome No similar effect in direct co‐culture with 3D engineered cartilage construct No agonist or antagonist assays were performed	[163]
2D compartmentalized with osteoclast secretome	Mice DRG and mice osteoclast	MEA chip	Osteoclasts secretome		n.a.		Increased firing of DRG exposed to osteoclasts secretome Osteoclast extracellular vesicles enhance axonal growth	[164]
3D compartmentalized innervated cancer spheroids	Mice DRG and human colorectal adenocarcinoma or human pancreatic carcinoma for the cancer spheroids	Capped microelectrodes	*Targeting ion channels*: Capsaicin	*Targeting receptors*: Bradykinin	n.a.		Tumor spheroids enhance sensitivity of nociceptor to stimuli	[135]
2D compartmentalized co‐culture with dendritic cells	Mice DRG and mice bone marrow‐derived dendritic cells	Calcium imaging	*Targeting ion channels*: Capsaicin viral (influenza A virus [IAV]) bacterial (UV‐inactivated Streptococcuspnneumoniae) fungal (Candidaalbicans) pathogens	*Targeting receptors*: IMQ (TLR‐7) Zymosan (TLR2 and Dectin‐1) polyinosinic: polycytidylie acid (TLR3) lipopolysaccharide (TLR4) flagellin (TLR5) CpG (TLR9)	*Targeting ion channels*: Lidocaine, QX314 (NaV)		Nociceptors agonist administration enhances the release of proinflammatory cytokines by dendritic cells Lidocaine inhibits cytokines production from dendritic cells CGRP released by nociceptors upregulate sentinel functions and inflammatory genes of dendritic cells	[165]
2D co‐culture with fibroblast‐like synoviocyte	Mice DRG and mice fibroblast‐like synoviocytes	Calcium imaging and patch‐clamp	*Targeting ion channels*: Capsaicin Menthol Cinnamaldehyde (TRPA1)		n.a.		Fibroblast‐like synoviocytes activated by TNF‐α enhance sensitivity of nociceptor to stimuli Enhanced TRPV1 function but downregulation of TRPA1 and TRPM8	[50]
3D Joint pain with 4 compartments (chondrocyte/osteoblast, synovial cells, adipose cells and sensory neurons)	n.a.	n.a.	n.a.		n.a.		No published study yet, only patent filling	[166] patent and [167]
2D co‐culture with astrocytes	hIPSCs nociceptors and astrocytes	MEA chip	*Targeting ion channels*: ATP AITC Capsaicin heat	*Targeting receptors*: TNF‐α	*Targeting ion channels*: Zonisamide, Phenytoin sodium Lamotrigine (NaV) Nimodipine, Levetiracetam, Nicardipine HCl (CaV)	*Targeting receptors*: APV (NMDAR) CNQX (AMPAR) FDA‐approved compounds for PNS and CNS diseases: Honokiol Capecitabine Rifampin Selumetinib Trametinib Everolimus VX‐745 Sorafenib Sorafenib tosylate (Different receptors)	Higher spontaneous activity over time in co‐culture All agonist except AITC increased the firing rate 10 out of 15 FDA‐approved compounds were	[168]
2D compartmentalized synapse of nociceptor‐DH neurons	Mice DRG and mice spinal cord DH neurons	Calcium imaging	Electrical stimulation		*Targeting receptors*: MK‐801 (NMDAR) CNQX (AMPAR)		Electrical stimulation of DRG cell bodies and axons sensitize DH neurons NMDA and AMPA receptor antagonist to DRG silences DH neuron responses,	[169] Similar studies: [126, 170, 171]
3D compartmentalized synapse of DRG and DH spheroids	Rat DRG and rat spinal cord DH neurons	Patch‐clamp Calcium imaging	*Targeting ion channels*: Capsaicin Optogenetic Stimulation Electrical Stimulation		*Targeting receptors*: CNQX (AMPAR) Bicuculline (GABA receptor) EDTA Lidocaine Clonidine (alpha‐2 adrenoceptor agonist that inhibit spinal receptors) Morphine		Stimulation of DRG results in a response in co‐cultured SCDH tissue through glutamatergic neurotransmission Lidocaine, clonidine and morphine have shown distinct electrophysiological profiles with lidocaine mainly desensitizes afferent pain fibers, while clonidine primarily impacts synaptic transmission.	[172]
3D compartmentalized innervated pancreatic pseudoislets and endometrium spheroids	hIPSC nociceptors, rat Schwann cells, rodent pancreatic pseudoislets and human endometrium spheroids	Patch‐clamp	*Targeting ion channels*: RTX (TRPV1) Hyperglycemia (45 mM glucose)		*Targeting ion channels*: CPZ (TRPV1)		When in co‐culture with Schwann cells, myelination of nociceptor was observed Myelin was damaged in hyperglycemia conditions but mitigated by epalrestat administration RTX exposure led to a release of substance P from nociceptors RTX induces neurite retraction, but when administered with CPZ, neurite remained unaffected Agonist and antagonist assays were performed without the pancreatic pseudoislets and human endometrium spheroids	[173] Similar study: [174]
3D compartmentalized co‐culture with Schwann cells and satellite glial cells	Rat explant DRG	Calcium imaging	*Targeting ion channels*: Capsaicin		n.a.		Immunostaining confirms presence of native non‐neuronal support cells Schwann cells and satellite glial cells in the rat DRG explants Electrophysiological characterization beyond the response to capsaicin is minimal	[175]
3D assembloid of 4 organoids: sensory organoids, DH spinal cord organoid, diencephalic organoids and cortical organoids	hIPSCs	Calcium imaging Patch‐clamp Neural probes	*Targeting ion channels*: Capsaicin TNP‐ and αβ‐MeATP	*Targeting receptors:* Glutamate Optogenetic stimulation	*Targeting receptors*: NBQX, APV		Neurons in assembloid showed synchronous wave‐like activity across all organoid regions Optogenetic stimulation and αβ‐MeATP exposure increased firing in all organoid regions of assembloids, but only in sensory organoids when organoids were non‐assembled but in close contact Assembloids with knockout and gain‐of‐function pathogenic SCN9A (encoding for NaV1.7) variants in sensory organoids were established Synchronous activity across the four organoids decreased in SCN9A knockout assembloid and increased in SCN9A gain‐of‐function assembloid	[176]

As pain sensitivity primarily relies on nociceptors, many research teams have focused on 2D monocultures of nociceptors to better understand their function (Table 4). In these studies, both agonists and antagonists target nociceptor‐specific ion channels and receptors, mainly the TRP family (such as TRPV1, TRPA1, TRPM8) as well as voltage‐gated channels like NaV but also GPCR and glutamate receptors. In addition to administering artificial compounds, one study examined the effect of inflammatory conditions on nociceptor sensitivity to pain stimuli. This was achieved by incubating mouse knee sensory neurons with real synovial fluid from a human OA patient, which increased both the spontaneous firing of these neurons and their excitability to the agonists [145]. Studies have also developed 3D models using iPSC‐derived nociceptors seeded at high density on ultra‐low‐cell adhesion surfaces [129, 146]. After generating these organoids, they were seeded in wells and exhibited axonal outgrowth in PDMS microchannels for axonal guidance, with confirmed expression of functional nociceptor‐specific ion channels and receptors. Further, unidirectional axon guidance [146], and even bidirectional guidance with two lateral compartments can be achieved [129]. This last study exploited the CMOS MEA technology and showed that with this high‐resolution platform they could detect single axonal AP and cluster nociceptors based on their waveforms into four different archetypes, each potentially representing a different nociceptor subtype [129].

Several studies have focused on the role of different KV and NaV channels (NaV1.7, NaV1.8, and NaV1.9) in DRG neurons for pain signaling (Table 4) [71, 147, 148, 149, 151]. They address how mutations or variations in these channels contribute to hyperexcitability in DRG neurons, leading to chronic pain conditions such as inherited erythromelalgia (IEM) and painful peripheral neuropathy. Pioneering experiments using rodent DRG neurons incorporated human channel mutations modeled via dynamic clamp techniques [177, 178, 179]. With the advent of iPSC technology, their research advanced to studies using patient‐derived iPSCs where dynamic clamp, voltage clamp, and current clamp were employed to analyze the functional properties of these channels and their impact on neuron excitability. With this setup, gain‐of‐function mutations could be identified by studies showing how these mutations enhance persistent currents, depolarize resting membrane potentials, and increase firing probabilities, thereby amplifying pain signals [148, 149, 151]. The obtained mechanistic insights into how specific channel mutations or conductance levels affect neuronal behavior further linked altered biophysical properties to increased excitability and pain phenotypes. The findings suggest potential therapeutic strategies, such as partial inhibition of identified NaV and KV channels, to mitigate hyperexcitability and pain.

While monocultures of nociceptors have been instrumental in advancing our understanding of pain mechanisms, they often lack the complexity of the native tissue environment. To address this limitation, researchers have increasingly turned to co‐culture systems that incorporate nociceptors with tissue‐specific cell types to replicate aspects of organ—or tissue pain sensitization (Table 5). These attempts have highlighted that nociceptors may exhibit altered receptor expression depending on the tissue target, leading to enhanced sensitivity of nociceptors, which can provide insights into how pain signals are modulated in response to the cellular microenvironment.

#### 5.3.1 Skin

Several co‐culture models of skin‐derived cells such as fibroblasts and/or keratinocytes and nociceptors have been described, depicting varying effects on neuronal responses and neurite outgrowth [153, 154, 155, 156, 157, 158, 159, 160, 161]. These models are not only relevant for biopharmaceutical companies but also for the cosmetics industry, as they help verify whether skincare active agents are non‐harmful and non‐painful for human consumers. Further, an artificial skin construct composed of fibroblasts embedded in hydrogel with keratinocytes, innervated with nociceptors, demonstrated that in the absence of the skin construct, neurite damage and retraction were more pronounced when capsaicin was administered, concluding that the skin constructs acted as a barrier by slowing the diffusion of capsaicin to the nociceptors (Table 5) [152]. Another study showed that upon stimuli administration, nociceptors in co‐culture with keratinocytes were more sensitive and active than those in monoculture, with a higher expression of TRPV1 [153].

#### 5.3.2 Vasculature

By co‐culturing endothelial cells and nociceptors, an elevated nociceptor sensitivity response to capsaicin as well as higher TRPV1 ion channel expression was shown, highlighting the critical role of vasculature on sensitizing nociceptors (Table 5). In co‐culture, larger vascular lumens and denser neurite outgrowth were also observed, implying that there is bidirectional crosstalk between the two cellular populations [162].

#### 5.3.3 Joint Derived Cells

Pain sensitization remains a key feature in OA pathophysiology, and co‐cultures of nociceptors with joint‐derived cells are critical in the context of OA‐related pain (Table 5). Additional co‐regulators include joint inflammation and articular cartilage deterioration. One study successfully co‐cultured nociceptors with fibroblast‐like synoviocytes (FLS) activated by the inflammatory cytokine TNF‐α, demonstrating increased neuronal sensitization by calcium imaging compared to nociceptor monocultures, highlighting the essential role of synovial tissue cells in pain modulation within the context of OA [50]. Another study established a compartmentalized innervated cartilage model composed of chondrocytes encapsulated in hydrogel, replicating the pro‐inflammatory joint microenvironment by administering human pro‐inflammatory M1 macrophage secretome to the model. Interestingly, it was observed that nociceptors exhibited enhanced axonal growth when exposed to the pro‐inflammatory secretome alone, but this effect was not replicated in direct co‐culture with the 3D engineered cartilage construct that was exposed for 48 h to M1 macrophage secretome. These findings suggest that differences in local concentration and stability of soluble mediators, and potentially a role in the dynamic cell–cell communication, may affect pain sensitization [163]. Furthermore, a more complex joint‐on‐chip model with three essential cellular compartments of the joint connected was developed, including a cartilage osteochondral complex, synovial tissue, and adipose tissue [167, 180]. Next, a fourth compartment of nociceptors innervating each of the compartments via microchannels to model OA pain was included [166]. This technology has remained in patent status, and no published studies have been conducted so far.

By implementing a MEA chip with PDMS‐based microfluidics to study the effects of osteoclasts in bone disease on the pain sensation, administration of osteoclast secretome on the axonal compartment demonstrated an enhanced outgrowth of sensory neurons, as well as an increased firing rate when exposed to osteoclasts secretome [164].

#### 5.3.4 Immune Cells

To explore the nociceptor‐immune crosstalk, a compartmentalized co‐culture model of dendritic cells and nociceptors was developed (Table 5) [165]. Upon agonist administration, nociceptors induced the release of proinflammatory cytokines (IL‐12 and IL‐6) by dendritic cells through direct physical contact. However, when treated with the NaV channel antagonist lidocaine, there was no enhanced cytokine production from dendritic cells. Interestingly, they also observed that nociceptors spread their AP to dendritic cells via calcium imaging. Additionally, the study showed that the neuropeptide CGRP released by nociceptors induces the upregulation of genes implicated in dendritic cells' sentinel functions and inflammatory responses, further highlighting the importance of immune‐nociceptor crosstalk [165].

#### 5.3.5 Cancer

A study cultured nociceptors in a 3D hydrogel scaffold with an NGF chemoattracting gradient, enabling them to innervate a compartment of cancer spheroids via glass‐designed microchannels (Table 5) [135]. When co‐cultured with cancer spheroids, an increased firing of nociceptors was observed upon administration of capsaicin and bradykinin, demonstrating the impact of cancer on pain sensitization.

#### 5.3.6 Glial Cells

Most co‐culture studies have focused solely on the modeling of the transduction step in the PNS between peripheral tissue cells and nociceptors, while glia and other non‐neuronal cells have important functions in the CNS and are known to be involved in neuroinflammatory processes and be contributors to persistent pain. Thus co‐cultures of neurons and glial cells represent valuable tools to study cellular mechanisms of nociception (Table 5) [181]. For this, a non‐compartmentalized co‐culture model of iPSC‐derived astrocytes and nociceptors on a MEA chip enabled the observation of more stable and higher spontaneous activity over time compared to monocultures, highlighting the role of astrocytes in modulating nociceptive signals and addressing the lack of (electro)maturity in iPSC‐derived nociceptor cultures. The model was challenged with agonists (capsaicin, allyl isothiocyanate [AITC], ATP, and 42°C heat) as well as the inflammatory mediator TNF‐α where all except AITC resulted in an elevated firing rate. Additionally, the researchers screened FDA‐approved compounds labeled for other PNS and CNS diseases, demonstrating that their model can be used to identify potential analgesic candidates from compound libraries. The efficacy of the compounds was measured using a Z' assay quality metric, a statistical parameter for evaluating high‐throughput screening assays [182], leading to 10 out of 15 compounds being identified as “hits.”

#### 5.3.7 DH‐Neurons

Some studies have also modeled the transmission step, namely the synapse between nociceptors and second‐order DH neurons of the spinal cord (Table 5) [169, 172, 183]. One study developed a three‐compartment PDMS co‐culture system involving mouse DRG and DH neurons [169]. The middle compartment contained DRG cell bodies, while the side compartments housed DRG axons and DH neurons, respectively. Electrical stimulation of the DRG cell bodies and axons resulted in increased electrical activity in DH neurons, as observed through calcium imaging, indicating that the synapse is functional. The administration of NMDAR and AMPAR antagonists in the DH compartment silenced DH neuron responses, suggesting that signal transmission via glutamate release is indeed maintained in vitro. Additionally, axotomy, that is, axon damage, was performed in the DRG axon compartment, and subsequent electrical stimulation led to increased firing of DH neurons. The same research group with the same cellular model has shown that administering NaV1.7 and NaV1.8 antagonists to the DRG neurons reduces synaptic transmission, but that is not the case when DRG axons are axotomized [183]. These findings suggest that changes in the excitability of sensory neurons following axotomy enhance synaptic transmission and the excitability of DH neurons, highlighting the importance of the DRG‐DH neuron axis in pain signaling.

Taking it a step further, a groundbreaking study established a circuit of four organoids aggregated into an assembloid, mimicking the pain pathway in embryos (Table 5). This pathway includes transmission from nociceptors to DH neurons, further connecting to thalamic neurons to simulate signal relay to the brain, and finally to cortical neurons where pain is processed and perceived [176]. They demonstrated synchronous wave‐like activity across all organoid regions, both spontaneously and in response to stimuli. Using CRISPR‐mediated gene editing, they created'disease mode' assembloids with knockout and gain‐of‐function pathogenic SCN9A (encoding for NaV1.7) variants in sensory organoids. In SCN9A knockout assembloids, the four organoids still displayed electrical activity but were no longer synchronized, while SCN9A gain‐of‐function assembloids exhibited hypersynchrony. These results highlight the capability to capture emergent and dysfunction properties that cannot be seen in single organoids, thus opening up the possibility of addressing disease pathophysiology at a circuit level [176].

For research groups with limited capacity to develop their own models, companies exist that offer in vitro pain model products with compartmentalized sensory neurons cultured on classical MEA chips with 48 electrodes. The cells express specific markers and functional activity, including thermoreactive properties and responsiveness to pain stimulus compounds [184]. The market availability of more complex and physiologically relevant co‐culture models remains limited. Apart from innervated skin models [154, 155], there is still no widely available co‐culture model of nociceptive circuits, leaving a gap for more representative pain models in the market. This is likely due to the scientific readiness of such models not yet being fully established.

By examining the interactions between nociceptors and various cell types in co‐culture models, researchers can gain insights into how pain signals are modulated dynamically in different cellular microenvironments. These models are invaluable for studying the molecular mechanisms underlying pain and for developing potential therapeutic interventions. As research continues to evolve, co‐culture and compartmentalized models will remain essential tools in the quest to decode the complexities of pain and improve pain management strategies.

## 6 Outlook

While the field of pain sensitization research has evolved significantly in the past years due to the development of innovative technologies, there is still a large unmet need to further improve our mechanistic understanding and tools for human relevant research and drug testing. The nociceptive pain phenomenon involves a diverse array of cell types, whereas current models have mainly focused on single‐cell systems of nociceptors, and some on two‐cell co‐cultures but very few on higher complexities [166, 175].

These in vitro models can still be considered limited in replicating the complexities of human pain pathways. To overcome this, advancement towards replicating more complex parts of the nociceptive circuit is needed. As an example, there is a crosstalk between nociceptors with non‐neuronal cells of the peripheral tissue and the CNS via the mediated release of pro‐/anti‐inflammatory mediators and neuropeptides, but additional physiological properties such as fluid flow, mechanical stimulation, aging, and co‐morbidities further affect the pain sensitization pathway. Therefore, incorporating more cellular interactions and a biomimetic microenvironment can lead to emergent properties, making realistic cell–cell interactions crucial for more accurate predictions of drug effects.

An ideal model would include mimicking cellular interactions during the transduction step between nociceptors and non‐neuronal cells from peripheral tissue, as well as the transmission step between nociceptors and second‐order neurons and non‐neuronal cells in the spinal cord within one platform. To our knowledge, no studies have developed such a model, likely due to the multitude of challenges involved, including optimizing a culture medium, managing cells that proliferate with nociceptors that do not, and building the right compartmentalized system to maintain a controlled environment. However, an ideal model should also be reproducible. Incorporating more complexity increases the risk of variability between cultures, making the model less predictive. Therefore, all culture protocols for different cell populations must be well optimized to ensure robustness and to closely match the functional and genetic characteristics of their corresponding primary cells.

Transitioning to more representative 3D models is highly desirable, as demonstrated by the innovative work of Kim et al. in their report of a four‐organoid assembloid [176]. Ideally, these models would involve complex organoids/spheroids that mimic the spatial configuration of cells within nociceptive circuits. For example, two organoids could replicate respectively the cellular populations of peripheral tissue and the spinal cord, both connected by nociceptor axons organized in 3D bundles, like in vivo conditions. However, these 3D cellular models can have inconsistent sizes, shapes, or structures, so the reproducibility challenge must be addressed [185]. Interfacing these 3D models presents challenges as well, such as the need for performant 3D MEAs (which are currently limited) instead of traditional planar MEA chips.

Adding additional sensors to the current system that usually only contains electrophysiology sensors allows us to acquire critical complementary informations that would aid our understanding of pain sensing phenomena. Multiparametric biosensing is essential for obtaining a deeper understanding of the complex nociceptive processes; for this, existing biosensors can be integrated [186, 187] with pro‐or anti‐inflammatory mediators to track them at interfaces between nociceptor axons and non‐neuronal cell populations, allowing for richer readouts.

iPSCs have revolutionized human pain research, and future endeavors will allow patient‐specific and/or gene variant mutation models by patient‐derived nociceptors in combination with additional key cell populations to address the human heterogeneity of pain sensing. For instance, a nociceptor monoculture derived from a patient with chronic neuropathic pain was developed and tested with lacosamide, an FDA‐approved compound for the treatment of seizures. Its off‐label use as an analgesic in these in vitro tests showed efficacy, and upon administration, the patient reported reduced pain according to their self‐assessment [188]. Extending this type of study to co‐culture models would be even more informative about the specificity of a patient's nociceptive circuitry. This approach opens numerous future opportunities for conducting “clinical trials‐on‐chip”. By stratifying patients suffering from different pain pathologies, with different ages, genders (acknowledging known gender differences in pain sensitivity and responses to pain drugs [189]) or ethnicities into subgroups according to the electrophysiological readouts and ideally readouts from other biosensors of their cultured nociceptive circuits by using high‐resolution technology like CMOS MEA chip. This would allow biopharmaceutical companies to screen more targeted pain medications, ensuring the right concentration of the right drug for different types of patients. Ultimately, in vitro pain models are not just tools for drug screening, they can enhance our mechanistic understanding of multicellular interactions within the human pain circuit and help decode its physiological complexity. With continued innovation, leveraging these models may 1 day help liberate billions from the burden of chronic pain.

## Author Contributions

**Dara Khosrowshahi:** writing – original draft preparation. **Liesbet Lagae:** supervision and writing – review and editing. **Johanna Bolander:** supervision, funding acquisition, writing – original draft preparation and writing – review and editing. All authors were involved in drafting and/or revising the manuscript.

## Conflicts of Interest

The authors declare no conflicts of interest.

## Data Availability

The authors have nothing to report.
